# Ultraprocessed Food Consumption and Risk of All-Cause Mortality in the Atherosclerosis Risk in Communities Study (ARIC)

**DOI:** 10.1016/j.cdnut.2026.109554

**Published:** 2026-10-12

**Authors:** Wanjin Guan, Jiaqi Yang, Shutong Du, Valerie K Sullivan, Casey M Rebholz

**Affiliations:** 1 Department of Epidemiology, Johns Hopkins Bloomberg School of Public Health, Baltimore, MD, United States; 2 Welch Center for Prevention, Epidemiology, and Clinical Research, Johns Hopkins University, Baltimore, MD, United States

**Keywords:** dietary intake, ultraprocessed food, food processing, Nova classification, all-cause mortality

## Abstract

**Background:**

Ultraprocessed food (UPF) consumption has increased substantially in the United States and globally, driven by its convenience, palatability, and aggressive marketing. Although previous studies have linked UPF consumption with adverse cardiometabolic outcomes, evidence on its relationship with mortality remains limited, particularly in racially and geographically diverse United States populations.

**Objectives:**

To examine associations of total UPF and UPF subtypes intake with all-cause mortality.

**Methods:**

The study included 11,185 participants (mean age 54 y; 55% female; 24% Black) from the Atherosclerosis Risk in Communities study at baseline (1987–1989). Diet was assessed using a validated 66-item food frequency questionnaire. Foods were classified according to the Nova classification system based on their degree of processing. UPF was measured in servings per day and assessed categorically and continuously. All-cause mortality was ascertained through 31 December 2022. We used multivariable Cox proportional hazards models to evaluate total UPF intake and intake of 9 UPF subgroups in relation to all-cause mortality.

**Results:**

Over a median follow-up of 30 y (301,093 person-years), 6656 deaths occurred. No significant association was observed between the highest and lowest quartiles of UPF intake and all-cause mortality [hazard ratio: 0.99; 95% confidence interval (CI): 0.92, 1.06). The highest quartile of consumption of ultraprocessed meats and spirits was associated with an 11% (95% CI: 1%, 21%) and 12% (95% CI: 2%, 23%) higher risk of all-cause mortality compared with the lowest quartile, respectively. In contrast, the highest quartile consumption of cereals was associated with a 13% (95% CI: 4%, 20%) lower risk of mortality.

**Conclusions:**

In this large prospective cohort of United States adults, overall UPF intake is not associated with all-cause mortality, although heterogeneous associations are observed with specific types of UPF. These findings highlight differences across UPF categories and warrant further investigation rather than treating UPF as a uniform exposure.

## Introduction

Consumption of ultraprocessed food (UPF) has increased substantially in recent decades [1], [2], [3], [4], accounting for 40%–60% of daily energy intake in the United States [5] and other countries [6], [7]. According to the Nova food classification, UPF refers to foods that are primarily or entirely produced from a series of industrial processes including hydrogenation, hydrolysis, extrusion, and molding, with hardly any intact food [8], [9]. Compared with minimally processed or unprocessed foods, UPFs typically contain more refined carbohydrates, saturated fat, sodium, and sugar and are characterized by poor nutritional quality, high energy density, low fiber content, and low micronutrient levels [10]. However, UPFs encompass diverse products, including processed meats, sweetened beverages, cereals, baked goods, and dairy desserts, which differ in their nutrient composition and processing characteristics [11]. Analyses of total UPF intake may therefore mask differing associations across these subtypes, highlighting the importance of subgroup-specific evaluation.

Consumption of UPF is associated with adverse cardiometabolic health outcomes [8], [10], [12]. The high-heat and high-pressure processing techniques used in UPF production generate chemical compounds such as acrylic acid, acrylamide, and advanced glycation end products, which contribute to increased oxidative stress, insulin resistance, and overall cardiometabolic worsening [13], [14]. Furthermore, chemical substances used during the food packaging process, like phthalates and bisphenol A, may also negatively impact the endocrine system [15]. Indeed, large-scale cohort studies have found that UPF consumption is associated with risk of diabetes, cardiovascular disease, and mortality [16], [17], [18].

Although longitudinal studies conducted in other countries have provided evidence on the health risks associated with UPF consumption, their findings may not fully extrapolate to the geographically and demographically diverse population of the United States [19]. In the United States, although several large-scale studies have examined the health impacts of UPF intake, important gaps remain, such as associations with mortality over extended follow-up and the role of specific UPF groups, particularly in diverse populations [20], [21], [22]. To address this gap, we conducted a prospective analysis in a racially and geographically diverse United States cohort to evaluate the long-term association between UPF consumption and all-cause mortality.

## Methods

### Study population

We obtained data from the Atherosclerosis Risk in Communities (ARIC) study, which has been thoroughly described before [23]. Briefly, it is a community-based prospective cohort study conducted in 4 sites in the United States and started in 1987, with a total enrollment of 15,792 predominantly Black and White participants. All participants gave written informed consent at each visit, and the study was approved by the institutional review boards at all study sites [23].

In this study, we excluded participants with >10 missing items from the food frequency questionnaire (FFQ) (*n* = 35) and those with implausible caloric intake (defined as women with total energy intake <500 or >3600 kcal and men with total energy intake <600 or >4200 kcal) or missing energy intake information (*n* = 154). We further excluded participants with missing covariates (*n* = 900), a history of major chronic diseases, including chronic kidney disease (CKD) (*n* = 181), heart disease (coronary heart disease, stroke, or heart failure) (*n* = 1372), diabetes (*n* = 1318), or cancer (*n* = 647) (Supplemental Figure 1). After these exclusions, a total of 11,185 participants were deemed eligible for subsequent analysis.

### Dietary assessment and food processing classification

Participants completed a 66-item semiquantitative FFQ at visit 1 (1987–1989) and 3 (1993–1995), which was a modified version of the Willett/Harvard FFQ [24]. Trained interviewers collected information on participants’ usual intake of foods and beverages over the past year. To help participants better estimate portion sizes, different-sized cups and glasses were used. To improve estimates of habitual dietary intake, we modeled dietary intake as a time-varying cumulative mean [25]. Visit 1 intake was assigned to person-time from visit 1 until visit 3. From the participant-specific visit 3 date onward, intake was updated to the cumulative average of visits 1 and 3. Participants who died or were censored before visit 3 contributed person-time only using visit 1 dietary data. For participants without a valid visit 3 dietary assessment, visit 1 dietary data were carried forward. Total energy intake was updated using the same approach.

We used the Nova classification system to categorize each food item into 1 of 4 groups: *1*) unprocessed or minimally processed foods, which are foods in their natural state or those that have undergone basic processing; *2*) processed culinary ingredients, defined as single components obtained from unprocessed or minimally processed foods (e.g., salt, sugar, vegetable oils, butter); *3*) processed foods, made by combining minimally processed or unprocessed foods and processed culinary ingredients; *4*) UPF, referring to industrial products primarily made from food derivatives and additives [8], [9]. We classified UPFs into the following subgroups: *1*) sugar-sweetened and artificially sweetened beverages, *2*) ultraprocessed meats, *3*) sugary snacks, *4*) fried foods, *5*) margarine, *6*) cereals, *7*) spirits (liquors), *8*) baked goods, and *9*) ice cream [26]. These subgroups were intended to represent commonly recognized food categories rather than nutritional composition. Classification was conducted independently by 2 reviewers and discrepancies were resolved by discussion and consensus with a third reviewer. The classification of individual FFQ food items according to the Nova system is provided in Supplemental Table 1. A sensitivity analysis was conducted after reclassifying items that were ambiguous, that is, orange or grapefruit juice was reclassified from ultraprocessed to unprocessed or minimally processed, and cooked cereals, including oatmeal, grits, and cream of wheat, were reclassified from processed to ultraprocessed.

We calculated the food intake within each Nova category in servings per day and adjusted for total energy intake using the residual method [24]. We regressed UPF servings per day on total energy intake and added the mean UPF intake to the resulting residuals, thereby recentering intake to the expected value at the mean total energy intake. This procedure was applied separately to visit 1 intake and the updated cumulative average intake using their corresponding energy measures. Dietary intake was relatively stable on average from visit 1 to visit 3 in the ARIC study [27].

### Outcome

The outcome of interest was all-cause mortality, with follow-up through 31 December 2022. Deaths were ascertained using a multifaceted approach, including hospital records, contact with proxies, or vital statistics from the National Death Index. All deaths were confirmed by death certificates, and the date was verified.

### Covariates

Sociodemographic data (age, sex, race, education level), smoking status, physical activity, and alcohol consumption were collected using questionnaires [28], [29]. Race center was defined as a composite variable capturing the joint distribution of race and ARIC field center. Race was collapsed into White and non-White categories, with participants from smaller racial groups included in the non-White category. Participants’ height and weight were measured by trained personnel and used to compute BMI (kg/m^2^) [18]. BMI categories were defined as normal weight (BMI <25 kg/m^2^), overweight (25 to <30 kg/m^2^), and obese (BMI ≥30 kg/m^2^). Area deprivation index (ADI) was calculated from participants’ addresses using the Singh method [30], [31]. Hypertension was defined as either self-reported use of blood pressure-lowering medication, systolic blood pressure ≥140 mm Hg, or diastolic blood pressure ≥90 mm Hg [18], [29]. Total serum cholesterol was determined using an enzymatic assay with a single aqueous reagent [18], [32]. Estimated glomerular filtration rate (eGFR) was calculated using the CKD Epidemiology Collaboration (CKD-EPI) creatinine race-free 2021 equation [33]. Covariates were selected a priori using the disjunctive cause criterion, guided by a directed acyclic graph (Supplemental Figure 2) [34].

### Statistical analyses

Baseline characteristics were summarized according to quartiles of energy-adjusted UPF consumption. Continuous variables were presented as mean ± SD, and categorical variables were presented as percentages.

Cox proportional hazards models were used to calculate hazard ratios (HRs) and 95% confidence intervals (CIs) for the association between UPF consumption and all-cause mortality. The proportional hazards assumption was assessed using Schoenfeld residuals, and there was no statistical evidence that this assumption was violated (*P* = 0.37). Model 1 was adjusted for age, sex, race center, and total energy intake. Model 2 (main model) was additionally adjusted for education level, ADI, smoking status, physical activity, and drinking status. Model 3 was further adjusted for BMI, hypertension status, total cholesterol level, and kidney function (assessed using eGFR, modeled as linear spline terms with a knot at 90 mL/min/1.73 m^2^) [35]. We examined UPF intake both categorically (quartiles of energy-adjusted intake) and continuously (per 1 additional daily serving). We evaluated the trend across UPF intake quartiles using median intake per quartile. The shape of the association was depicted using a restricted cubic spline with 4 knots placed at the 5th, 35th, 65th, and 95th percentiles.

We assessed associations between each of 9 UPF subgroups and all-cause mortality. Energy-adjusted intakes for each food group were divided into quartiles. HRs for each food group were calculated with adjustment for Model 2 covariates and simultaneous inclusion of all 9 UPF food groups. Total UPF intake was not included in the model.

We performed substitution analyses to estimate the impact of replacing UPF with minimally and unprocessed alternatives on risk of all-cause mortality, as well as substituting 1 serving of ultraprocessed meat with 1 serving of cereal [36]. This substitution analysis of UPF subgroups was selected post hoc to further assess the heterogeneity between types of UPF and to quantify the potential impact of replacing one type of UPF with another type of food with an opposing direction of association with mortality. Relevant food groups were included simultaneously in units of servings/day, and substitution HRs were calculated as exp(β_replacement − β_replaced), with 95% CIs derived from the corresponding variance-covariance estimates.

To explore potential heterogeneity across demographic, metabolic, and neighborhood socioeconomic contexts, we also performed stratified analyses based on the following subgroups: age (above compared with below 60 y), sex (male and female), BMI (normal weight, overweight, and obesity), and ADI (above compared with below median). Interactions were tested by adding multiplicative interaction terms to the Cox models and evaluating significance using the likelihood ratio test. The UPF subgroup, stratified, and substitution analyses were considered secondary and exploratory analyses.

We conducted sensitivity analyses by *1*) reclassifying ambiguous food items, *2*) excluding deaths occurring within the first 5 y of follow-up to reduce potential reverse causation, and *3*) using multiple imputation by chained equations to assess the impact of missing covariate data. Under the missing-at-random assumption, 50 imputed datasets were generated using the exposure, outcome, cumulative hazard, and all model covariates, and estimates were combined using Rubin’s rules.

All analyses were conducted using R software, version 4.4.2 (http://www.R-project.org/). A 2-sided *P* value <0.05 was considered statistically significant.

## Results

### Baseline characteristics

The mean baseline age of participants was 54 y, 45% were men, 24% were Black, and the mean BMI was 27 kg/m^2^ (Table 1). Participants consumed a median of 6 (25th percentile: 5, 75th percentile: 7) servings/d of UPF. Participants in the highest quartile consumed 8 servings of UPF per day on average. Compared with the first quartile (lowest UPF consumption), participants with the highest consumption of UPF tended to be White, former smoker, current drinker, and have a lower ADI score. Main food groups contributing to UPF intake were sugar- and artificially sweetened beverages (25%), followed by baked goods (21%), margarine (17%), and ultraprocessed meats (11%) (Figure 1). Cereals, fried foods, sugary snacks, spirits, and ice cream collectively comprised 27% of total UPF consumption. Across quartiles of UPF intake, beverages consistently contributed the largest proportion and increased from 23% in quartile 1 to 27% in quartile 4, whereas meats and cereals showed modest declines, from 15% to 9% and from 7% to 6%, respectively. Participants excluded because of missing covariates were more likely to be Black and to have hypertension, to have higher BMI and ADI, to have lower education and physical activity levels, and had similar serum cholesterol and energy intake than those included in the analytic sample (Supplemental Table 2).

**TABLE 1:** Baseline participant characteristics for the overall study population and according to quartile of frequency of ultraprocessed food intake

	Overall	Quartile 1	Quartile 2	Quartile 3	Quartile 4
*n*	11,185	2797	2796	2796	2796
Ultraprocessed food intake (servings/d) median (25th, 75th percentile)	5.70 [4.68, 6.89]	3.92 [3.21, 4.35]	5.22 [4.96, 5.47]	6.24 [5.96, 6.53]	7.96 [7.33, 9.03]
Deaths (%)	6656 (59.5)	1730 (61.9)	1681 (60.1)	1646 (58.9)	1599 (57.2)
Age (y)	53.7 (5.7)	53.8 (5.7)	53.7 (5.7)	53.6 (5.7)	53.5 (5.6)
Sex: Male	4997 (44.7)	1300 (46.5)	1163 (41.6)	1205 (43.1)	1329 (47.5)
Black (%)	2637 (23.6)	852 (30.5)	788 (28.2)	657 (23.5)	340 (12.2)
BMI (kg/m^2^)	27.1 (5.0)	26.9 (5.0)	27.0 (5.0)	27.2 (5.0)	27.4 (5.0)
BMI category (%)					
Normal weight, <25.0 kg/m^2^	4072 (36.4)	1053 (37.6)	1061 (37.9)	1019 (36.4)	939 (33.6)
Overweight, 25–<30.0 kg/m^2^	4501 (40.2)	1135 (40.6)	1098 (39.3)	1114 (39.8)	1154 (41.3)
Obesity, ≥30.0 kg/m^2^	2612 (23.4)	609 (21.8)	637 (22.8)	663 (23.7)	703 (25.1)
Alcohol status (%)					
Current drinker	6679 (59.7)	1630 (58.3)	1606 (57.4)	1643 (58.8)	1800 (64.4)
Former drinker	1888 (16.9)	474 (16.9)	468 (16.7)	463 (16.6)	483 (17.3)
Never drinker	2618 (23.4)	693 (24.8)	722 (25.8)	690 (24.7)	513 (18.3)
Education level (%)					
Less than completed high school	2292 (20.5)	637 (22.8)	599 (21.4)	554 (19.8)	502 (18.0)
High school	3683 (32.9)	838 (30.0)	896 (32.0)	966 (34.5)	983 (35.2)
At least some college	5210 (46.6)	1322 (47.3)	1301 (46.5)	1276 (45.6)	1311 (46.9)
Smoking status (%)					
Current smoker	2892 (25.9)	795 (28.4)	712 (25.5)	688 (24.6)	697 (24.9)
Former smoker	3544 (31.7)	843 (30.1)	885 (31.7)	856 (30.6)	960 (34.3)
Never smoker	4749 (42.5)	1159 (41.4)	1199 (42.9)	1252 (44.8)	1139 (40.7)
Physical activity score^1^	2.5 (0.8)	2.5 (0.8)	2.4 (0.8)	2.5 (0.8)	2.5 (0.8)
Hypertension (%)	3173 (28.4)	816 (29.2)	827 (29.6)	782 (28.0)	748 (26.8)
eGFR (mL/min/1.73 m^2^)	103 (11.1)	103 (11.2)	103 (11.3)	103 (11.3)	103 (10.7)
Total serum cholesterol (mg/dL)	213.7 (40.9)	213.3 (42.1)	213.8 (40.3)	214.3 (40.6)	212.6 (40.4)
Total energy intake (kcal)	1632 (634.7)	1777 (663.7)	1475 (559.6)	1521 (570.4)	1754 (677.9)
ADI score	46.4 (22.0)	49.2 (22.9)	48.2 (22.6)	46.6 (21.9)	41.6 (19.6)

Baseline participant characteristics for the overall study population and according to quartile of frequency of ultraprocessed food intake are reported as mean (SD) for continuous variables and number (%) for categorical variables.

Abbreviations: ADI, area deprivation index; eGFR, estimated glomerular filtration rate.

1 Physical activity score for sport-related exercise during leisure time (range 1–5).

**FIGURE 1:**
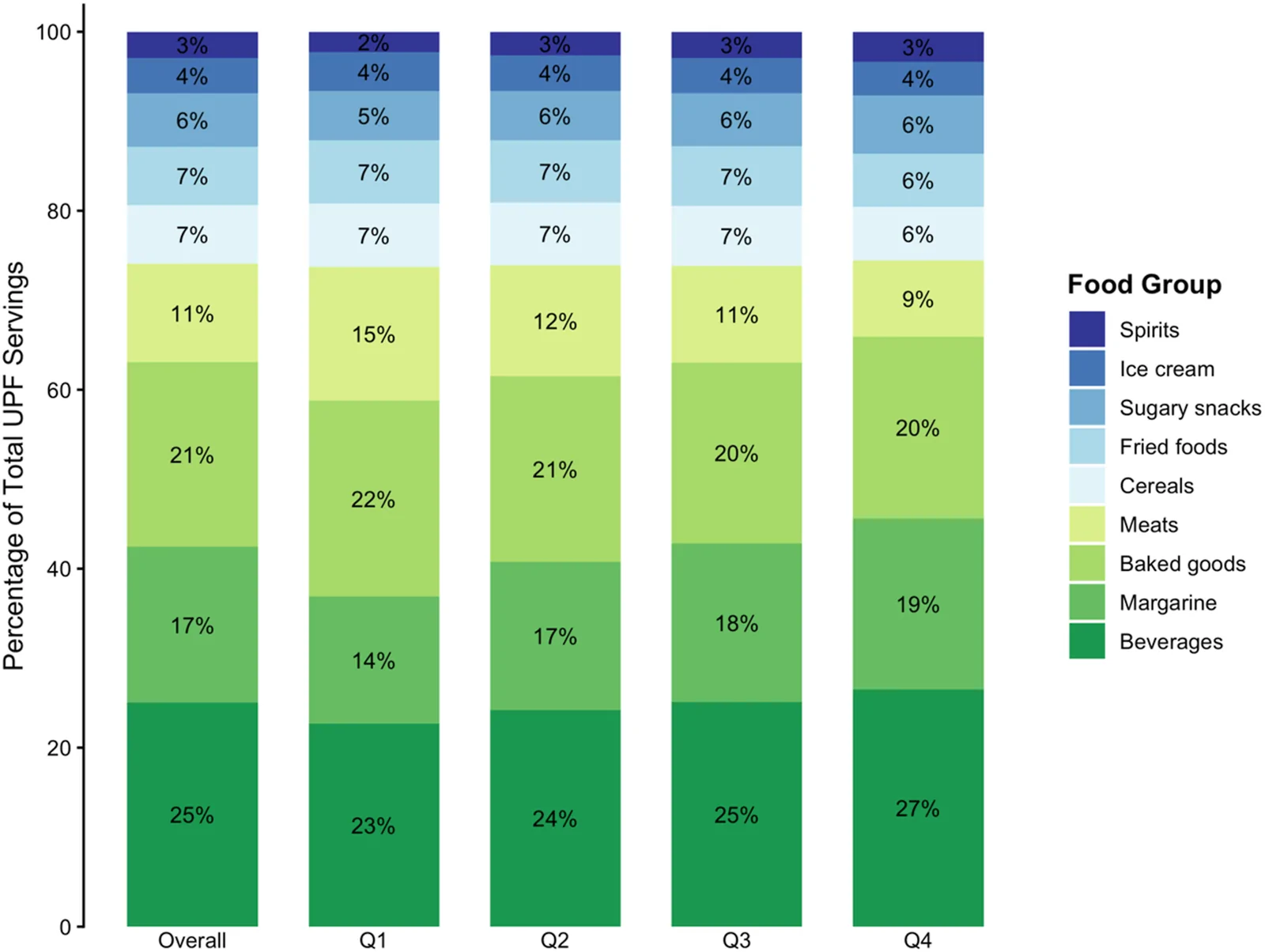
Distribution of ultraprocessed food consumption across 9 food groups overall and within each quartile of UPF intake. Food groups included beverages, baked goods, margarine, meats, cereals, sugary snacks, fried foods, spirits, and ice cream. UPF, ultraprocessed food.

### Association between UPF intake and all-cause mortality

During follow-up (301,093 person-years, median follow-up time 30 y, 25th–75th percentile: 22–33 y), 6656 deaths occurred. UPF intake was not significantly associated with all-cause mortality across quartiles and in the continuous analysis in models 1, 2, and 3 (Table 2). There was a nonlinear association between UPF and mortality, with the lowest mortality risk observed in the middle of the distribution of UPF consumption (Supplemental Figure 3). Replacing UPF with unprocessed or minimally processed food was not significantly associated with mortality risk (HR: 1.00; 95% CI: 0.99, 1.01).

**TABLE 2:** Risk of all-cause mortality associated with ultraprocessed food consumption, analyzed according to quartiles and continuously

	Quartile of UPF consumption					Continuous analysis	
	Quartile 1	Quartile 2	Quartile 3	Quartile 4	*P* for trend	Per 1 additional serving/d	*P* value
Mortality, *n* (%)	1740 (62.2)	1644 (58.8)	1614 (57.7)	1658 (59.3)			
HR (95% CI)							
Model 1^1^	Reference	0.96 (0.89, 1.02)	0.96 (0.90, 1.03)	1.01 (0.94, 1.08)	0.73	1.00 (0.99, 1.02)	0.53
Model 2^2^		0.94 (0.88, 1.01)	0.94 (0.88, 1.01)	0.99 (0.92, 1.06)	0.90	1.00 (0.99, 1.01)	0.95
Model 3^3^		0.94 (0.87, 1.00)	0.94 (0.87, 1.00)	0.97 (0.91, 1.04)	0.55	1.00 (0.98, 1.01)	0.62

Hazard ratios (HRs) and 95% CIs for all-cause mortality by quartiles and continuous intake of ultraprocessed food (servings/day).

Abbreviations: ADI, area deprivation index; CI, confidence interval; eGFR, estimated glomerular filtration rate; HR, hazard ratio.

1 Model 1 was adjusted for age, sex, race center, and total energy intake.

2 Model 2 was adjusted for education level, ADI, smoking status, physical activity score, and drinking status.

3 Model 3 was further adjusted for baseline BMI, hypertension status, total cholesterol, and kidney function (defined by eGFR level, modeled as 2 linear spline terms with a knot at 90 mL/min/1.73 m^2^).

### Associations between UPF subtypes and risk of all-cause mortality

Higher intake of cereal was associated with lower risk of all-cause mortality (Figure 2; Supplemental Table 3). Compared with participants in the lowest quartile, those in the highest quartile of cereal intake had a 13% lower risk of death (HR = 0.87; 95% CI: 0.80, 0.96; *P*-trend = 0.002). In contrast, higher intake of ultraprocessed meats was associated with an elevated mortality risk, with participants in the highest quartile experiencing an 11% higher risk of death compared with those in the lowest quartile (Model 2: HR = 1.11; 95% CI: 1.01, 1.21; *P*-trend = 0.04). A similar positive association was observed for spirits (HR = 1.12; 95% CI: 1.02, 1.23; *P*-trend < 0.001). No statistically significant associations were found for other UPF subgroups, including beverages, margarine, baked goods, fried foods, sugary snacks, and ice cream. In substitution analyses, the largest mortality risk reduction was observed when replacing 1 daily serving of ultraprocessed meats with 1 serving of ultraprocessed cereals (HR: 0.82; 95% CI: 0.75, 0.90) and when replacing spirits with cereals (HR: 0.82; 95% CI: 0.76, 0.88) (Supplemental Table 4). Results were consistent across models.

**FIGURE 2:**
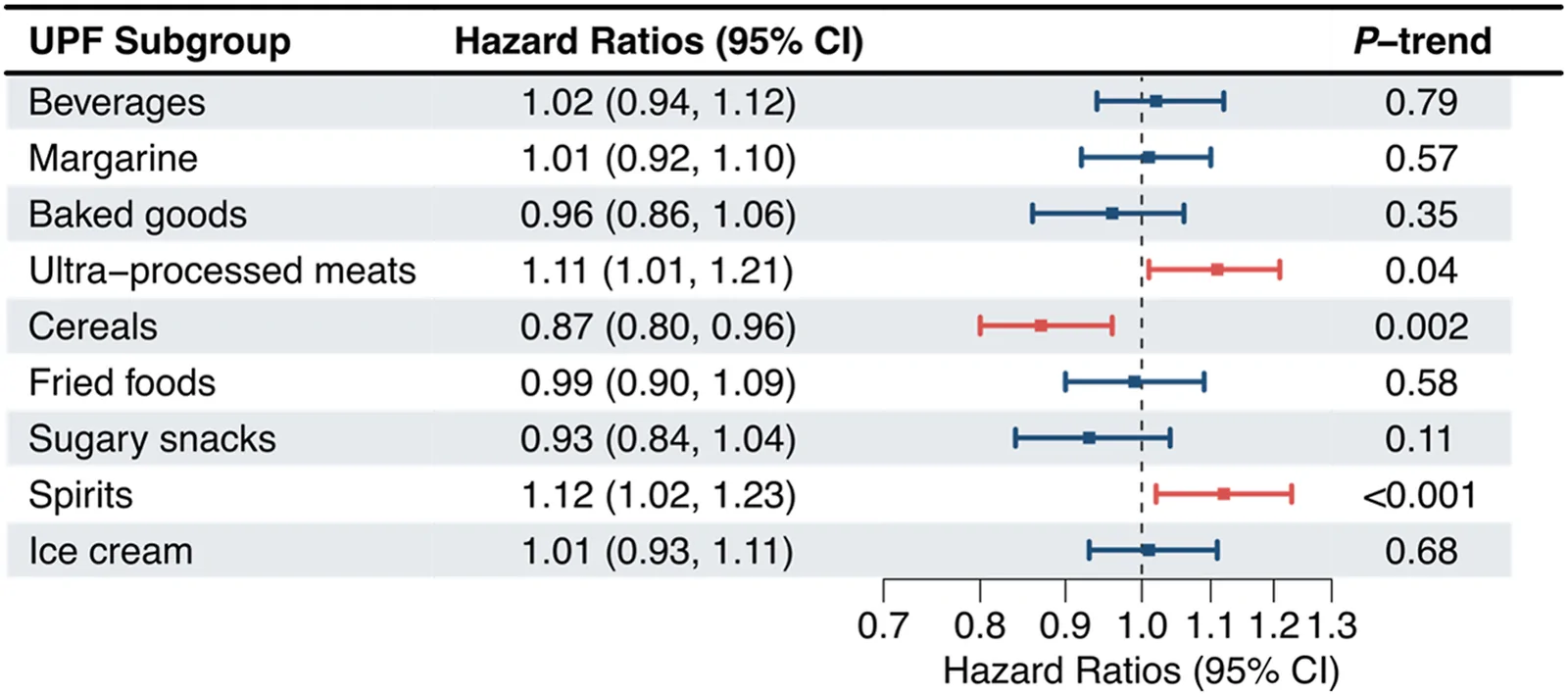
Risk of all-cause mortality for the highest vs. the lowest quartile of ultraprocessed food subtype consumption. Hazard ratios and 95% confidence intervals for all-cause mortality comparing the highest with the lowest quartile of ultraprocessed food intake frequency, measured in servings per day, adjusted for age, sex, race center, total energy intake, education level, ADI, smoking status, physical activity score, and drinking status. ADI, area deprivation index; CI, confidence interval; HR, hazard ratio.

### Subgroup analysis and sensitivity analysis

The association between UPF intake and all-cause mortality was broadly consistent across subgroups defined by sex (*P*-interaction = 0.45), BMI (*P*-interaction = 0.57), and ADI (*P*-interaction = 0.78) (Figure 3). There was statistical interaction by age (*P*-interaction = 0.02), with higher UPF consumption associated with higher risk of mortality among participants aged 60 y or older (HR: 1.05, 95% CI: 0.93, 1.19); however, associations were not statistically significant within either age subgroup.

**FIGURE 3:**
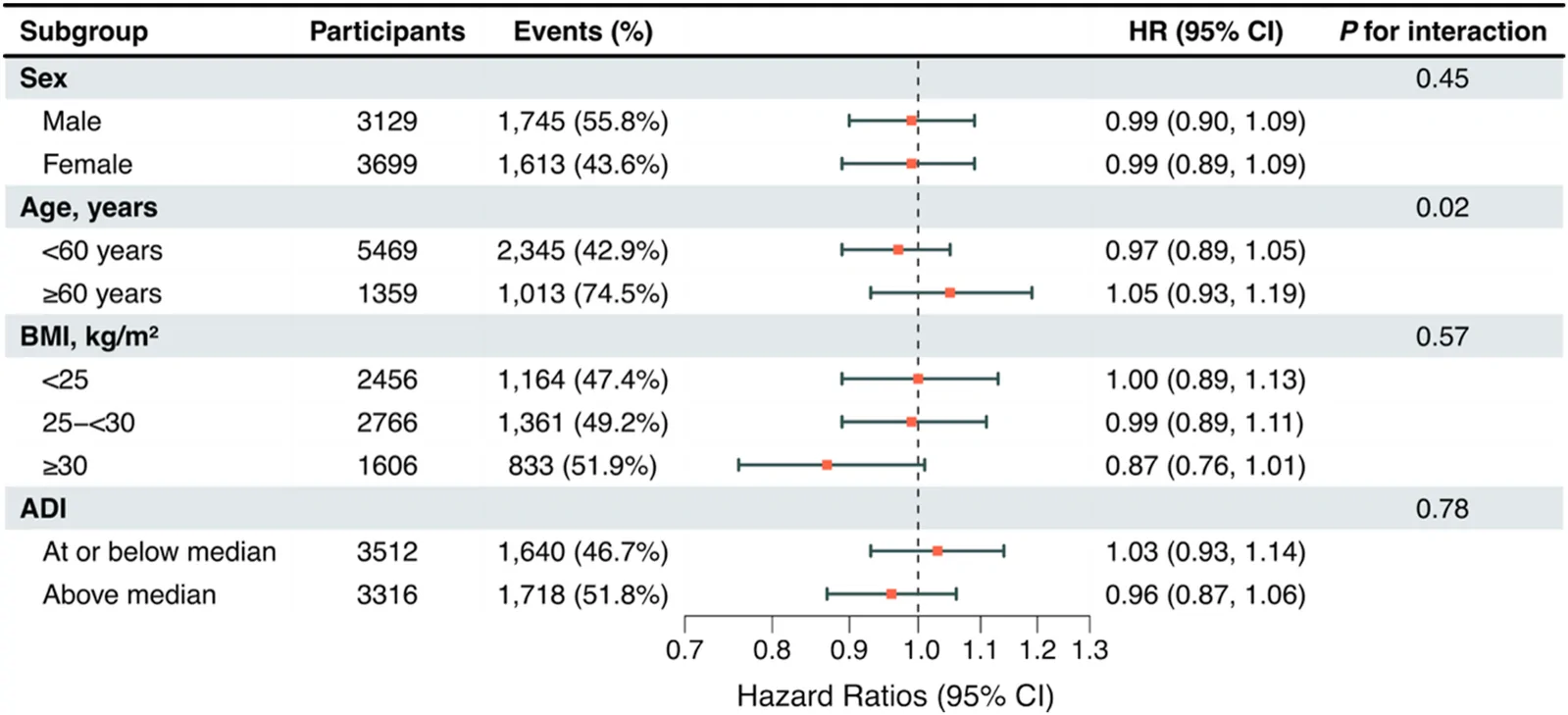
Association between ultraprocessed food consumption and all-cause mortality across subgroups of the study population. Hazard ratios (HRs) and 95% confidence intervals (CIs) were presented for highest quartile vs. lowest quartile of ultraprocessed food consumption. The model was adjusted for age, sex, race center, total energy intake, education level, ADI, smoking status, physical activity score, and drinking status. ADI, area deprivation index; CI, confidence interval; HR, hazard ratio.

After reclassifying ambiguous food items (Supplemental Table 5), excluding deaths occurring within the first 5 y of follow-up (Supplemental Table 6), and after multiple imputation for missing covariates (Supplemental Table 7), the results were consistent with the primary analysis, and the overall conclusions remained unchanged.

## Discussion

In this large prospective cohort study of over 11,000 middle-aged United States adults followed for 30+ y, we observed that overall UPF intake was not associated with all-cause mortality. Ultraprocessed meats and spirits were significantly associated with higher risk of all-cause mortality, whereas ultraprocessed cereals were inversely associated with risk of mortality.

The lack of a significant association between UPF intake and all-cause mortality in this study is consistent with some previous findings but contrasts with others [17], [37], [38], [39], [40], [41]. In populations including the Korean Genome and Epidemiology Study-Health Examinees cohort study, UPF intake was closely associated with higher socioeconomic status, urban residence, and greater access to healthcare [37]. These factors may be associated with lower mortality risk and have a greater impact on mortality than dietary exposure. Meanwhile, UPF consumption may coexist with other unhealthy lifestyle factors [42]. Although our analysis considered a series of demographic, behavioral, and clinical covariates, residual confounding factors cannot be excluded. UPF intake can still serve as a representative of unmeasured lifestyle and environmental variables, which can alter the true association with mortality.

Because the FFQ used to assess dietary intake was administered in the late 1980s and the mid-1990s, our exposure estimates reflect the food environment of that period, which differs substantially from that of today. These dietary assessments also preceded the development of the Nova classification, which was introduced in 2009, formally described as a 3-group classification in 2010, and subsequently refined into the current 4-group system [12], [43], [44]. Consequently, the 66-item ARIC FFQ was not originally designed to capture the brand names, ingredient lists, additives, and preparation details required for more granular Nova classification. At the time, most UPFs were shelf-stable and convenience-oriented products, such as packaged breads, canned soups, and breakfast cereals [45]. Although these items were processed, their ingredient lists were typically simpler and included fewer additives than current formulations [46]. In contrast, many contemporary UPFs incorporate novel emulsifiers, artificial sweeteners, colorants, and engineered flavor systems, which may have distinct metabolic and health effects [47], [48]. Consistent with these changes, national data indicate that UPF intake among United States adults increased from 53.5% of total energy intake in 2001–2002 to 57.0% in 2017–2018, accompanied by changes in the relative contributions of specific UPF groups [49]. Thus, dietary assessments from the earlier ARIC visits may not fully represent UPF consumption patterns later in follow-up or in the contemporary food environment. These temporal changes, together with the retrospective application of Nova to a historical FFQ, may have contributed to exposure misclassification and heterogeneity within the broad UPF subgroups. A recent study of 3 large United States prospective cohorts with ≤34 y of follow-up similarly reported only a weak association between UPF intake and all-cause mortality, with an HR of 1.04 (95% CI: 1.01, 1.06) comparing the highest with the lowest quartile of UPF consumption [38]. These findings suggest that temporal changes in product composition and availability, along with evolving individual-level factors such as age, employment, comorbidities, and household structure, may partly explain the null association observed in the study [50].

Another possible explanation could be the role of the food matrix. The food matrix refers to the complex physical and chemical assembly of nutrients and non-nutrient components within a food, which interact to influence the release, accessibility, digestibility, and stability of food compounds [51], [52]. Variations in the food matrix can lead to differential effects on nutrient absorption, postprandial glycemic responses, satiety signals, and hormonal pathways involved in metabolism [51]. The mechanical or thermal breakdown of cellular structures can facilitate the release of bioactive components such as carotenoids, polyphenols, glucosinolates, and vitamins [53]. Certain processing techniques, such as micellization to enhance carotenoid absorption, fruit homogenization to improve phytochemical bioavailability, and thermal isomerization to increase lycopene uptake, demonstrate that food processing can, under specific conditions, improve the accessibility and physiological effects of some nutrients and bioactive compounds [54], [55], [56]. However, in the case of UPF, these potential benefits often coexist with unfavorable characteristics such as poor nutritional quality, high energy density, and the widespread use of additives. This heterogeneity may be partially explained by the fact that not all UPFs are nutritionally equivalent, and that specific structural and compositional features of certain UPFs could attenuate the expected health risks in population studies.

The diverse ways in which processing alters food structure and metabolism, and the differential mortality risk associated with various UPF subtypes highlight the heterogeneous nature of UPFs and the limitations of treating them as a uniform exposure. Among UPF subgroups, ultraprocessed meats showed strong association with all-cause mortality. High-temperature processing and cooking can generate mutagenic iron compounds, N-nitroso compounds, and lipid peroxidation products, all of which have been implicated in carcinogenesis and cardiovascular toxicity [57], [58], [59], [60]. Thermal treatment may also lead to the formation of harmful neoformed contaminants such as acrolein, which have been associated with platelet activation, endothelial dysfunction, and increased cardiovascular disease risk [61]. In addition, red meat is a major source of heme iron and L-carnitine, the latter of which can be metabolized by gut microbiota into trimethylamine-N-oxide, a proatherogenic compound linked to cardiovascular disease [62]. Ultraprocessed red meat products also tend to contain a range of sodium-based additives, including sodium chloride, nitrites, and phosphates, that have been associated with elevated blood pressure and adverse cardiovascular outcomes.

We found that higher intake of cereals was associated with a lower risk of all-cause mortality. A plausible interpretation is that individuals may be consuming healthier versions, such as breakfast cereals fortified with essential micronutrients [63]. In a meta-analysis, higher breakfast cereal consumption was associated with a 15% lower risk of all-cause mortality [64]. Many cereals in the United States food supply are enriched with B vitamins, iron, and folic acid, which may positively contribute to overall nutrient intake, particularly among individuals with limited dietary quality [47]. These inverse associations may also partly reflect residual confounding or reverse causation. The FFQ provided limited information on specific product characteristics, such as the whole-grain or added-sugar content of cereals. Therefore, we could not determine whether these components of UPF contributed to the observed associations with cereals. Future research is necessary to evaluate causal relationships between types of UPF and mortality.

This study has several notable strengths. It was conducted within a well-characterized, community-based prospective cohort with >30 y of follow-up, allowing for robust temporal assessment of dietary exposures and mortality outcomes. Second, the cohort predominantly includes Black and White participants from 4 geographically diverse United States communities, allowing us to examine the associations across diverse regional settings. Third, we incorporated time-updated estimates of UPF intake by using repeated dietary assessments, which allowed for a more accurate representation of participants’ long-term dietary exposures. Finally, the ascertainment of all-cause mortality in the ARIC study was rigorous and standardized, reducing the likelihood of outcome misclassification. The consistency of our findings across different analytical models supports the robustness of the observed associations.

Nevertheless, several limitations should be acknowledged. First, because detailed information on food preparation methods and brand names was unavailable, misclassification of processing level is possible. At present, there is no gold standard for Nova-based classification, and we did not conduct a validation study for our categorization of UPF. To reduce potential misclassification, 2 independent reviewers classified all food items, with a third reviewer resolving discrepancies. Second, although the ARIC study collected comprehensive sociodemographic, clinical, and behavioral data, and we adjusted for a wide range of covariates, residual confounding remains a concern. Factors such as psychologic stress, occupational exposures, and income instability may influence both dietary behavior and mortality risk but were not fully characterized. Third, as with all observational studies, reverse causation is possible, particularly given the long follow-up period. To address this, we excluded participants with prevalent cardiovascular disease, diabetes, cancer, and CKD at baseline and conducted a sensitivity analysis excluding deaths that occurred within the first 5 y of follow-up. Fourth, although we incorporated the 2 dietary assessments in our exposure assessment, change in dietary intake over subsequent follow-up was not captured, although change in dietary intake in middle-aged adults in the ARIC study has been reported to be minimal [27]. The lack of dietary data over the entire study period may have attenuated the estimates of association.

In this large, community-based prospective cohort study, we did not observe a significant association between total UPF intake and all-cause mortality. However, associations varied across UPF subgroups. Higher intake of ultraprocessed meat was consistently associated with increased mortality risk, and higher intake of cereal was inversely associated with mortality. These findings highlight the importance of considering specific UPF categories rather than treating UPF as a uniform exposure and may inform future research to determine whether processing level, nutritional composition, or other food characteristics account for these associations. Modifying the types of UPFs consumed, for example, consuming cereal instead of meat, may increase longevity.

## Author contributions

The authors’ responsibilities were as follows – WG: wrote the manuscript and conducted analyses; CMR: designed the study and provided supervision; JY, SD, VKS, CMR: constructed the diet score; and all authors: contributed to interpretation of data, revised the manuscript critically, and approved the final manuscript.

## Data availability

The data are available upon application and approval from the NHLBI Biologic Specimen and Data Repository Information Coordinating Center (BioLINCC; https://biolincc.nhlbi.nih.gov/studies/aric/) or the ARIC Steering Committee. The codebook and analytic code will be provided upon reasonable request.

## Declaration of generative AI And AI-assisted technologies in the writing process

The authors declare that no generative AI or AI-assisted technologies were used in the writing of this manuscript.

## Funding

The ARIC study has been funded in whole or in part with federal funds from the National Heart, Lung, and Blood Institute, National Institutes of Health, Department of Health and Human Services, under contract nos. 75N92022D00001, 75N92022D00002, 75N92022D00003, 75N92022D00004, and 75N92022D00005. Metabolomics measurements were sponsored by the National Human Genome Research Institute (3U01HG004402-02S1). CMR was supported in part by a grant from the NHLBI (R01 HL153178).

## Conflict of interest

The authors report no conflicts of interest.
