# Supplementary material for: Ultraprocessed Food Consumption and Risk of All-Cause Mortality in the Atherosclerosis Risk in Communities Study (ARIC)
Source: Curr Dev Nutr. 2026 Oct 12;10(11):109554. doi: 10.1016/j.cdnut.2026.109554 (PMC13760103; doi:10.1016/j.cdnut.2026.109554)
Supplement: Supplementary file 1 [file mmc1.docx]

Supplemental Material

Ultra-processed food consumption and risk of all-cause mortality in the Atherosclerosis Risk in Communities Study (ARIC)

Wanjin Guan, Jiaqi Yang, Shutong Du, Valerie K. Sullivan, Casey M. Rebholz

Supplemental Figure 1. Flow chart for the selection of the analytic sample from the Atherosclerosis Risk in Communities (ARIC) Study.

Abbreviations: ADI, area deprivation index, BMI, body mass index; eGFR, estimated glomerular filtration rate, FFQ, food frequency questionnaire.

Supplemental Figure 2. Directed acyclic graph of the hypothesized causal relationships among ultra-processed food intake, covariates, and all-cause mortality

Abbreviations: ADI, area deprivation index, BMI, body mass index; eGFR, estimated glomerular filtration rate.

Supplemental Figure 3. Restricted cubic spline of the association between ultra-processed food consumption and risk of all-cause mortality.

The gray histogram displays the distribution of ultra-processed food consumption in the study population. The red solid line represents the adjusted hazard ratios (HRs) for all-cause mortality, modeled using restricted cubic splines with four knots placed at the 5th, 35th, 65th, and 95th percentiles of ultra-processed food intake. The reference value was set at the 25th percentile (e.g., 4.72 servings/day). The shading indicates the 95% confidence intervals (CIs). The model was adjusted for age, sex, race-center, total energy intake, education level, ADI, smoking status, physical activity score, and drinking status. Abbreviations: HR, hazard ratio; CI, confidence interval; ADI, area deprivation index.

Supplemental Table 1. Nova classification of food items included in the Atherosclerosis Risk in Communities Study food frequency questionnaire*

| **Nova food category** | | **Food items** |
| --- | --- | --- |
| Unprocessed or minimally processed foods | | Milk (whole, skim, or low-fat); yogurt; fresh, packaged, or frozen fruits and vegetables; beans and other legumes; fresh or frozen beef, pork, lamb, poultry, other meats, and fish; liver and other organ meats; eggs; homemade pie prepared from scratch; nuts without added sugar or salt; mashed or baked potatoes; spaghetti, noodles, and other pasta; foods fried at home; rice; coffee; tea |
| Processed culinary ingredients | | Butter; table sugar; plant oils; table salt |
| Processed foods | | Cheese (all types); bacon; canned tuna; cooked cereals (oatmeal, grits, or cream of wheat); white bread; dark or whole-grain bread; peanut butter; beer; wine |
| Ultra-processed foods | Sugar- and artificially sweetened beverages | Orange or grapefruit juice; low-calorie soft drinks; regular soft drinks; fruit-flavored punch or noncarbonated beverages (lemonade, Kool-Aid, or Hawaiian Punch) |
|  | Ultra-processed meats | Hamburgers; hot dogs; processed meats (sausage, salami, or bologna); beef, pork, or lamb in mixed dishes |
|  | Sugary snacks | Chocolate bars or pieces (Hershey’s, Plain M&M’s, Snickers, or Reese’s); non-chocolate candy |
|  | Fried foods | Potato or corn chips; French fries; foods fried away from home |
|  | Margarine | Margarine and other spreads |
|  | Cereals | Cold breakfast cereal |
|  | Spirits | Hard liquor |
|  | Baked goods | Chocolate bars or pieces; non-chocolate candy; ready-made pie; doughnuts; biscuits or cornbread; Danish pastries, sweet rolls, coffee cake, or croissants; cake or brownies; cookies |
|  | Ice cream | Ice cream |

Abbreviations: ARIC, Atherosclerosis Risk in Communities; FFQ, food frequency questionnaire. UPF subgroups were not mutually exclusive; chocolate and non-chocolate candy were included in both the sugary snacks and baked goods subgroups.

* Adapted with permission from reference # 26 (Du S, Kim H, Rebholz CM. Higher Ultra-Processed Food Consumption Is Associated with Increased Risk of Incident Coronary Artery Disease in the Atherosclerosis Risk in Communities Study. J Nutr. 2021;151:3746-54.)

Supplemental Table 2. Characteristics of participants included in the analytic sample and those excluded because of missing covariate data

|  | Analytic sample | Excluded due to missing covariates |
| --- | --- | --- |
| n | 11185 | 915 |
| Ultra-processed food intake, servings/d, median (25th, 75th percentile) | 5.7 [4.7, 6.9] | 5.4 [4.4, 6.6] |
| Deaths (%) | 6656 (59.5) | 616 (67.3) |
| Age, yrs | 53.7 (5.7) | 54.1 (5.8) |
| Male sex | 4997 (44.7) | 384 (42.0) |
| Black (%) | 2637 (23.6) | 426 (46.6) |
| BMI, kg/m^2^ | 27.1 (5.0) | 28.3 (5.7) |
| BMI category (%) |  |  |
| Normal weight, <25.0 kg/m^2^ | 4072 (36.4) | 260 (28.4) |
| Overweight, 25-<30.0 kg/m^2^ | 4501 (40.2) | 342 (37.4) |
| Obesity, ≥30.0 kg/m^2^ | 2612 (23.4) | 297 (32.5) |
| Alcohol status (%) |  |  |
| Current drinker | 6679 (59.7) | 377 (41.2) |
| Former drinker | 1888 (16.9) | 163 (17.8) |
| Never drinker | 2618 (23.4) | 315 (34.4) |
| Education level (%) |  |  |
| Less than completed high school | 2292 (20.5) | 283 (30.9) |
| High school | 3683 (32.9) | 259 (28.3) |
| At least some college | 5210 (46.6) | 348 (38.0) |
| Smoking status (%) |  |  |
| Current smoker | 2892 (25.9) | 247 (27.0) |
| Former smoker | 3544 (31.7) | 248 (27.1) |
| Never smoker | 4749 (42.5) | 406 (44.4) |
| Physical activity score^a^ | 2.5 (0.8) | 2.3 (0.8) |
| Hypertension (%) | 3173 (28.4) | 375 (41.0) |
| eGFR, ml/min/1.73 m^2^ | 102.8 (11.1) | 101.4 (14.0) |
| Total serum cholesterol, mg/dL | 213.7 (40.9) | 213.9 (42.7) |
| Total energy intake, kcal | 1632 (635) | 1615 (672) |
| ADI score | 446.4 (22.0) | 61.9 (25.3) |

Values are presented as mean (SD), median (25th, 75th percentile), or n (%). Percentages among participants excluded because of missing covariates were calculated using the number with available data for each characteristic. Abbreviations: ADI, Area Deprivation Index; BMI, body mass index; eGFR, estimated glomerular filtration rate; SD, standard deviation; UPF, ultra-processed food.

Supplemental Table 3. Risk of All-cause Mortality Associated with Quartiles of Ultra-processed Food Subtype Consumption

| Quartile of 9 UPF  subgroup intake | Mortality,  n (%) | Hazard ratios (95% CI) | | |
| --- | --- | --- | --- | --- |
|  |  | Model 1^a^ | Model 2^b^ | Model 3^c^ |
| **Beverages** |  |  |  |  |
| Quartile 1 (n=2,797) | 1,831 (65.5) | Reference | | |
| Quartile 2 (n=2,796) | 1,638 (58.6) | 0.92 (0.85, 1.01) | 0.96 (0.88, 1.05) | 0.95 (0.88, 1.04) |
| Quartile 3 (n=2,796) | 1,575 (56.3) | 0.87 (0.80, 0.95) | 0.91 (0.83, 0.99) | 0.89 (0.82, 0.97) |
| Quartile 4 (n=2,796) | 1,612 (57.7) | 0.98 (0.90, 1.07) | 1.02 (0.94, 1.12) | 0.98 (0.90, 1.07) |
| *P* value for trend |  | 0.59 | 0.79 | 0.51 |
| **Margarine** |  |  |  |  |
| Quartile 1 (n=2,797) | 1,716 (61.4) | Reference | | |
| Quartile 2 (n=2,796) | 1,611 (57.6) | 0.97 (0.89, 1.06) | 0.96 (0.88, 1.05) | 0.97 (0.89, 1.06) |
| Quartile 3 (n=2,796) | 1,641 (58.7) | 0.98 (0.90, 1.07) | 1.00 (0.91, 1.09) | 0.99 (0.90, 1.08) |
| Quartile 4 (n=2,796) | 1,688 (60.4) | 1.00 (0.91, 1.09) | 1.01 (0.92, 1.10) | 1.01 (0.93, 1.10) |
| *P* value for trend |  | 0.76 | 0.57 | 0.49 |
| **Baked goods** |  |  |  |  |
| Quartile 1 (n=2,797) | 1,776 (63.5) | Reference | | |
| Quartile 2 (n=2,796) | 1,657 (59.3) | 0.97 (0.89, 1.07) | 1.00 (0.91, 1.10) | 1.01 (0.92, 1.11) |
| Quartile 3 (n=2,796) | 1,597 (57.1) | 0.92 (0.83, 1.01) | 0.91 (0.82, 1.01) | 0.92 (0.83, 1.02) |
| Quartile 4 (n=2,796) | 1,626 (58.2) | 0.94 (0.84, 1.04) | 0.96 (0.86, 1.06) | 0.99 (0.89, 1.10) |
| *P* value for trend |  | 0.28 | 0.35 | 0.70 |
| **Meat** |  |  |  |  |
| Quartile 1 (n=2,796) | 1,656 (59.2) | Reference | | |
| Quartile 2 (n=2,796) | 1,603 (57.3) | 1.12 (1.03, 1.23) | 1.09 (0.99, 1.19) | 1.09 (1.00, 1.19) |
| Quartile 3 (n=2,796) | 1,650 (59.0) | 1.13 (1.03, 1.23) | 1.05 (0.96, 1.15) | 1.05 (0.96, 1.15) |
| Quartile 4 (n=2,796) | 1,746 (62.4) | 1.20 (1.09, 1.31) | 1.11 (1.01, 1.21) | 1.10 (1.00, 1.20) |
| *P* value for trend |  | <0.001 | 0.04 | 0.07 |
| **Cereals** |  |  |  |  |
| Quartile 1 (n=2,796) | 1,781 (63.7) | Reference | | |
| Quartile 2 (n=2,796) | 1,580 (56.5) | 0.87 (0.80, 0.95) | 0.95 (0.87, 1.03) | 0.94 (0.86, 1.02) |
| Quartile 3 (n=2,796) | 1,686 (60.3) | 0.76 (0.70, 0.83) | 0.88 (0.80, 0.96) | 0.88 (0.80, 0.96) |
| Quartile 4 (n=2,796) | 1,608 (57.5) | 0.71 (0.65, 0.78) | 0.87 (0.80, 0.96) | 0.89 (0.81, 0.97) |
| *P* value for trend |  | <0.001 | 0.002 | 0.005 |
| **Fried foods** |  |  |  |  |
| Quartile 1 (n=2,797) | 1,774 (63.4) | Reference | | |
| Quartile 2 (n=2,796) | 1,674 (59.9) | 1.07 (0.98, 1.17) | 1.03 (0.94, 1.13) | 1.02 (0.94, 1.12) |
| Quartile 3 (n=2,796) | 1,589 (56.8) | 1.00 (0.91, 1.10) | 0.97 (0.88, 1.07) | 0.96 (0.87, 1.05) |
| Quartile 4 (n=2,796) | 1,619 (57.9) | 1.02 (0.93, 1.12) | 0.99 (0.90, 1.09) | 1.00 (0.91, 1.10) |
| *P* value for trend |  | 0.92 | 0.58 | 0.76 |
| **Sugary snacks** |  |  |  |  |
| Quartile 1 (n=2,796) | 1,762 (63.0) | Reference | | |
| Quartile 2 (n=2,796) | 1,640 (58.7) | 0.94 (0.86, 1.03) | 0.99 (0.90, 1.08) | 1.00 (0.91, 1.09) |
| Quartile 3 (n=2,796) | 1,641 (58.7) | 0.90 (0.81, 0.99) | 0.90 (0.82, 1.00) | 0.91 (0.82, 1.00) |
| Quartile 4 (n=2,796) | 1,612 (57.7) | 0.94 (0.85, 1.05) | 0.93 (0.84, 1.04) | 0.94 (0.84, 1.05) |
| *P* value for trend |  | 0.22 | 0.11 | 0.12 |
| **Spirits** |  |  |  |  |
| Quartile 1 (n=1,672) | 1,023 (61.2) | Reference | | |
| Quartile 2 (n=1,672) | 915 (54.7) | 0.97 (0.88, 1.07) | 0.97 (0.88, 1.07) | 0.96 (0.87, 1.06) |
| Quartile 3 (n=1,672) | 914 (54.7) | 1.01 (0.91, 1.12) | 1.00 (0.90, 1.11) | 1.00 (0.90, 1.11) |
| Quartile 4 (n=1,672) | 1,114 (66.6) | 1.17 (1.07, 1.28) | 1.12 (1.02, 1.23) | 1.10 (1.00, 1.21) |
| *P* value for trend |  | <0.001 | <0.001 | <0.001 |
| **Ice cream** |  |  |  |  |
| Quartile 1 (n=2,796) | 1,727 (61.8) | Reference | | |
| Quartile 2 (n=2,796) | 1,617 (57.8) | 1.05 (0.96, 1.16) | 1.07 (0.97, 1.17) | 1.07 (0.98, 1.18) |
| Quartile 3 (n=2,795) | 1,583 (56.6) | 1.06 (0.96, 1.16) | 1.06 (0.97, 1.17) | 1.07 (0.97, 1.18) |
| Quartile 4 (n=2,795) | 1,727 (61.8) | 0.99 (0.90, 1.08) | 1.01 (0.93, 1.11) | 1.02 (0.93, 1.11) |
| *P* value for trend |  | 0.28 | 0.68 | 0.74 |

Hazard ratios (HR) and 95% CI for all-cause mortality, according to quartile of frequency of ultra-processed food intake (servings/day).

Model 1^a^ was adjusted for age, sex, race-center, and total energy intake.

Model 2^b^ was additionally adjusted for education level, ADI, smoking status, physical activity score, and drinking status.

Model 3^c^ was further adjusted for baseline BMI, hypertension status, total cholesterol, and kidney function (defined by eGFR level, modeled as two linear spline terms with a knot at 90 mL/min/1.73 m^2^).

Abbreviations: HR, hazard ratio; CI, confidence interval; BMI, body mass index; ADI, area deprivation index, eGFR, estimated glomerular filtration rate.

Supplemental Table 4. Associations of replacing one serving per day of ultra-processed meat or spirits with other ultra-processed food subgroups with all-cause mortality

| **Replaced group** | **Replacement group** | **Hazard ratios (95% CI)** | ***P* value** |
| --- | --- | --- | --- |
| Ultra-processed meat | beverages | 0.94 (0.87, 1.01) | 0.11 |
|  | margarine | 0.94 (0.87, 1.02) | 0.14 |
|  | baked goods | 0.90 (0.83, 0.98) | 0.01 |
|  | cereals | 0.82 (0.75, 0.90) | <0.001 |
|  | fried foods | 0.89 (0.79, 1.01) | 0.07 |
|  | sugary snacks | 0.92 (0.82, 1.03) | 0.15 |
|  | spirits | 1.01 (0.93, 1.09) | 0.86 |
|  | ice cream | 0.95 (0.85, 1.06) | 0.39 |
| Spirits | beverages | 0.94 (0.89, 0.98) | 0.004 |
|  | margarine | 0.94 (0.89, 0.98) | 0.007 |
|  | baked goods | 0.90 (0.85, 0.95) | <0.001 |
|  | ultra-processed meat | 0.99 (0.92, 1.07) | 0.86 |
|  | cereals | 0.82 (0.76, 0.88) | <0.001 |
|  | fried foods | 0.89 (0.81, 0.98) | 0.01 |
|  | sugary snacks | 0.92 (0.83, 1.01) | 0.07 |
|  | ice cream | 0.95 (0.86, 1.04) | 0.25 |

Models were adjusted for age, sex, race-center, education level, Area Deprivation Index, smoking status, physical activity, alcohol use, and total energy intake. Abbreviations: ADI, Area Deprivation Index; CI, confidence interval; HR, hazard ratio; UPF, ultra-processed food.

Supplemental Table 5．Hazard ratios (HRs) and 95% CIs for all-cause mortality by quartiles and continuous intake of reclassified ultra-processed food (servings/day)*

| Quartile of UPF consumption | Categorical analysis | | | | |  | Continuous analysis | |
| --- | --- | --- | --- | --- | --- | --- | --- | --- |
|  | Quartile 1 | Quartile 2 | Quartile 3 | Quartile 4 | *P* for trend |  | Per 1 additional  serving/d | *P*-value |
| Mortality, n (% in total) | 1,725 (61.7) | 1,686 (60.3) | 1,635 (58.5) | 1,610 (57.6) |  |  |  |  |
| Hazard ratios (95% CI) |  |  |  |  |  |  |  |  |
| Model1^a^ | Reference | 0.95 (0.89, 1.02) | 0.96 (0.89, 1.03) | 1.01 (0.94, 1.08) | 0.57 |  | 1.01 (0.99, 1.02) | 0.39 |
| Model2^b^ |  | 0.95 (0.89, 1.02) | 0.93 (0.87, 1.00) | 0.98 (0.91, 1.05) | 0.58 |  | 1.00 (0.98, 1.01) | 0.69 |
| Model3^c^ |  | 0.96 (0.90, 1.03) | 0.93 (0.87, 1.00) | 0.97 (0.90, 1.04) | 0.32 |  | 1.00 (0.98, 1.01) | 0.47 |

Model 1^a^ was adjusted for age, sex, race-center, and total energy intake.

Model 2^b^ was adjusted for education level, ADI, smoking status, physical activity score, and drinking status.

Model 3^c^ was further adjusted for baseline BMI, hypertension status, total cholesterol, and kidney function (defined by eGFR level, modeled as two linear spline terms with a knot at 90 mL/min/1.73 m^2^).

*For the sensitivity analysis, orange or grapefruit juice was reclassified from ultra-processed to unprocessed or minimally processed, and cooked cereals, including oatmeal, grits, and cream of wheat, were reclassified from processed to ultra-processed.

**Abbreviations:** HR, hazard ratio; CI, confidence interval; BMI, body mass index; ADI, area deprivation index, eGFR, estimated glomerular filtration rate.

Supplemental Table 6. Hazard ratios (HRs) and 95% CIs for all-cause mortality by quartiles and continuous intake of ultra-processed food (servings/day) after excluding participants who died in the first 5 years of follow-up.

| Quartile of UPF consumption | Categorical analysis | | | | |  | Continuous analysis | |
| --- | --- | --- | --- | --- | --- | --- | --- | --- |
|  | Quartile 1 | Quartile 2 | Quartile 3 | Quartile 4 | *P* for trend |  | Per 1 additional  serving/d | *P*-value |
| Mortality, n (% in total) | 1,649 (60.7) | 1,629 (59.4) | 1,608 (58.3) | 1,558 (56.6) |  |  |  |  |
| Hazard ratios (95% CI) |  |  |  |  |  |  |  |  |
| Model1^a^ | Reference | 0.97 (0.91, 1.04) | 0.98 (0.92, 1.06) | 1.03 (0.96, 1.10) | 0.31 |  | 1.01 (0.99, 1.02) | 0.23 |
| Model2^b^ |  | 0.96 (0.89, 1.03) | 0.96 (0.90, 1.03) | 1.01 (0.94, 1.08) | 0.60 |  | 1.00 (0.99, 1.02) | 0.62 |
| Model3^c^ |  | 0.95 (0.89, 1.02) | 0.96 (0.89, 1.03) | 0.99 (0.93, 1.07) | 0.97 |  | 1.00 (0.99, 1.01) | 0.96 |

Model 1^a^ was adjusted for age, sex, race-center, and total energy intake.

Model 2^b^ was adjusted for education level, ADI, smoking status, physical activity score, and drinking status.

Model 3^c^ was further adjusted for baseline BMI, hypertension status, total cholesterol, and kidney function (defined by eGFR level, modeled as two linear spline terms with a knot at 90 mL/min/1.73 m^2^).

**Abbreviations:** HR, hazard ratio; CI, confidence interval; BMI, body mass index; ADI, area deprivation index, eGFR, estimated glomerular filtration rate.

Supplemental Table 7. Complete-case and multiple-imputation analyses of ultra-processed food intake and all-cause mortality

| Analysis | Quartile 1 | Quartile 2 | Quartile 3 | Quartile 4 | P for trend | Per 1 additional serving/d | P-value |
| --- | --- | --- | --- | --- | --- | --- | --- |
| Complete-case analysis | Reference | 0.94 (0.88, 1.01) | 0.94 (0.88, 1.01) | 0.99 (0.92, 1.06) | 0.90 | 1.00 (0.99, 1.01) | 0.95 |
| Multiple-imputation analysis | Reference | 0.93 (0.87, 0.99) | 0.93 (0.87, 1.00) | 0.98 (0.91, 1.05) | 0.64 | 1.00 (0.99, 1.01) | 0.77 |

Values are hazard ratios (95% confidence intervals). Models were adjusted for age, sex, race-center, education level, Area Deprivation Index, smoking status, physical activity, alcohol use, and total energy intake. Tests for trend were conducted by assigning participants the median UPF intake of their quartile and modeling this value continuously. Multiple-imputation estimates were combined across 50 imputed datasets using Rubin’s rules.

Abbreviations: CI, confidence interval; HR, hazard ratio; UPF, ultra-processed food.
